# Problematic familial alcohol use and heavy episodic drinking among upper secondary students: a moderator analysis of teacher-rated school ethos

**DOI:** 10.1186/s13104-021-05773-8

**Published:** 2021-09-22

**Authors:** Gabriella Olsson, Sara Brolin Låftman, Joakim Wahlström, Bitte Modin

**Affiliations:** Department of Public Health Sciences, Centre for Health Equity Studies (CHESS), Stockholm University, 10691 Stockholm, Sweden

**Keywords:** Binge drinking, Adolescents, School, Effective schools, Contextual

## Abstract

**Objective:**

Building on findings reported in a previous publication, the objective of this study is to explore if teacher-rated indicators of school ethos modify the association between problematic familial alcohol use and heavy episodic drinking among upper secondary students. Data were based on combined information from two separate surveys conducted in 2016 among 4709 students and 1061 teachers in 46 Stockholm upper secondary schools, with linked school-level information from administrative registers. Multilevel binary logistic regression analyses were performed.

**Results:**

Problematic familial alcohol use was associated with an increased likelihood of heavy episodic drinking among upper secondary students (OR 1.36, 95% CI 1.12–1.65). Cross-level interactions revealed that the association was weaker among students attending schools with higher levels of teacher-rated ethos. This was true for overall school ethos (OR 0.79, 95% CI 0.65–0.97) and for four of five studied sub-dimensions of ethos: staff stability (OR 0.78, 95% CI 0.65–0.95); teacher morale (OR 0.79, 95% CI 0.65–0.97); student focus (OR 0.80, 95% CI 0.65–0.97); and academic atmosphere (OR 0.79, 95% CI 0.65–0.96). The sub-dimension “structure and order for dealing with unwanted behaviour” did however not moderate the association between problematic familial alcohol use and heavy episodic drinking (OR 0.95, 95% CI 0.77–1.18).

## Introduction

Heavy episodic drinking (HED), often defined as consuming at least six alcoholic drinks on one occasion at least monthly [1], during adolescence has been linked to injuries [2] and a range of negative social and behavioural outcomes in youth [3] and in adulthood [4]. Among those with early onset [5] and in more vulnerable populations [4, 6], the risk for long-term consequences seems to be particularly elevated. A key risk factor for excessive drinking in youth is problematic familial alcohol use; both in terms of serious and long-term parental alcohol problems [7] and parental drinking at lower levels of risk drinking [8, 9]. Modelling of familial behaviours, impaired parenting practices [10], but also inherited psychological and biological personality traits that in interaction with the environmental stressors in the family put individuals at greater risk of developing problematic drinking are some of the suggested mechanisms [7].

Previous research concerned with the impact of problematic drinking in the family on youth alcohol consumption [8, 9] has mainly sought for risk and protective factors in the context of the family, but more rarely explored how conditions in the family interact with conditions in other social and institutional settings. Yet, it is reasonable to assume that such interacting effects exists [11]. Research into school effects does suggest that conditions in school are linked to student risk behaviours, including alcohol consumption [12–14]. In addition, school effectiveness research has long showed that qualities of the school per se matter for students’ academic performance and behaviours, regardless of the students’ own background [15]. Yet most of the research conducted in this field has been concerned with the direct effect of conditions in school on youth behaviours, while less research has been devoted to exploring the compensatory role of schools. Existing studies do however suggest that this is a relevant inquiry that ought to be further explored [16–18].

The current study builds on, and extends, the analyses reported in a study by Olsson et al. [19] which was based on the assumption that conditions in school may buffer against problematic conditions in the family. Olsson et al. [19] examined the association between familial problematic drinking and heavy drinking among ninth grade students (age 15–16 years) and, more specifically, the moderating role of schools’ level of teacher-reported student focus. Student focus can be understood as one sub-dimension of school ethos that refers to positive teacher-student relationships. In line with previous research [9, 10, 20–22], Olsson et al. [19] reported that there was a positive association between familial problematic drinking and heavy drinking among students. However, the association was weaker among students attending schools with higher teacher ratings of student focus, suggesting a protective effect.

The present paper is based on the same data material as the study by Olsson et al. [19], but extends it in two ways, namely by analysing the association between problematic familial alcohol use and offspring drinking among older students, and by examining the potentially moderating role of teacher-rated school ethos in a broader sense.

The aim of the current study is to explore if teacher-rated indicators of school ethos modify the association between problematic familial alcohol use and heavy episodic drinking among upper secondary students, focusing both on an overall measure of school ethos and on five different sub-dimensions of school ethos: (1) staff stability; (2) teacher morale; (3) structure and order for dealing with unwanted behavior; (4) student focus; and (5) academic atmosphere [23]. Building on the result of the study by us [19], two hypotheses were formulated:

H1. Problematic drinking in the family is associated with increased likelihood of heavy episodic drinking in the offspring.

H2. A strong teacher rated school ethos may moderate this association.

## Main text

### Data material and methods

The data was drawn from two cross-sectional surveys performed in 2016: the Stockholm School Survey (SSS) and the Stockholm Teacher Survey (STS), which were combined. In addition, school-level official register information from the Swedish National Agency for Education has been merged with the data.

The SSS is carried out every other year by Stockholm municipality among students in grade 9 of compulsory school (ages 15–16 years) and in grade 2 of upper secondary school (ages 17–18 years) in all public schools and in a large number of independent schools in Stockholm. Students complete the questionnaires in the classroom. The response rate for the 2016 survey has been estimated to 78 per cent [24].

The STS was performed among teachers in 2014 and in 2016 as part of a research project at Stockholm University. Teachers in schools whose students took part in the SSS were invited to participate in a web survey. In 2014, the STS was performed among teachers in the senior-level schools that took part in the SSS, and in 2016 it was performed among teachers in both senior-level and upper secondary schools that participated in the SSS. School-level measures of teacher ratings of, e.g., school ethos and school leadership were formed by taking the mean values of each school. These measures were subsequently linked to the SSS student level data. The response rate among upper secondary teachers was 58% [23, 25].

The present study was based on data from 2016: the SSS collected among students in the second grade of upper secondary school (17–18 years), school-level information from the STS, and official register information on schools from the Swedish National Agency for Education. The study sample includes responses from 4709 students and 1061 teachers distributed across 46 upper secondary schools. More information on the data material is found elsewhere [23, 25].

### Ethics

According to a decision by the Regional Ethical Review Board of Stockholm (2010/241-31/5), data from the Stockholm School Survey are not subject to consideration for ethical approval since the questionnaires are completed anonymously with no information on personal identification. The Regional Ethical Review Board of Stockholm has granted ethical approval for the Stockholm Teacher Survey (2015/1827-31/5).

### Measures

Heavy episodic drinking was assessed by one question in the SSS: “How often do you drink the following amounts of alcohol at any one time: 18 cl spirits or a whole bottle of wine or four large bottles of strong cider/alcopop or four cans of class III beer or six cans of class II beer?”. The response categories were “Do not drink alcohol”, “Never”, “Very seldom”, “A few times each year”, “A few times a month”, “A couple of times a month”, and “A few times a week”. The item was dichotomised by classifying students who answered “A few times a month” or more often as engaging in heavy episodic drinking. This is an established measure, used in alcohol surveys among youth in Sweden since the 1970s [24, 26] and in previous research [27, 28]. It is deemed roughly equivalent [28] to WHO [1] definition of HED as consuming at least 60 g or more of pure alcohol on at least one occasion in the past 30 days. A consumption of 60 g of pure alcohol corresponds approximately to five standard alcoholic drinks [1].

Problematic familial alcohol use was measured by one question in the SSS: “Do you think someone in your family drinks too much alcohol?” The response categories were “Yes”, “No”, and “Don’t know”. Those who answered “Don’t know” were excluded from the analysis. The same question was used in the study by Olsson et al. [19].

School ethos was captured by an index of 17 items (Cronbach’s alpha  =  0.94) [25]. This overall measure of ethos was also divided into five sub-dimensions: (1) staff stability, referring to the level of sick-leave among teachers, staff turnover, and the frequency of substitute teachers at the school (3 items, Cronbach’s alpha  =  0.55); (2) teacher morale, referring to whether the teachers have a strong work ethic, work with great enthusiasm, take pride in their school, and feel confident as classroom leaders (4 items; Cronbach’s alpha  =  0.93); (3) structure and order for dealing with unwanted behaviour, referring to the schools’ value system, whether the school actively works on issues such as violence, bullying and harassment among students, whether teachers feel confident about what they may and may not do if violent situations arise among students, and whether the rules for order and conduct are clear at the school (4 items, Cronbach’s alpha  =  0.92); (4) student focus among teachers, referring to teachers’ positive feedback to, and high expectations of, the students, whether the teachers take time with students even if they want to discuss something other than schoolwork, and whether the students are treated with respect (4 items, Cronbach’s alpha  =  0.79); and (5) academic atmosphere, referring to the extent to which the school provides a stimulating learning environment and whether the students’ motivation is a stimulating part of work (2 items, Cronbach’s alpha  =  0.89). The measures of overall school ethos and of the five sub-dimensions were all standardised (mean  =  0; standard deviation  =  1). The measures have been used in prior research [23].

A number of control variables capturing sociodemographic characteristics were also included. At the student level, we controlled for gender, family structure, parental university education, and migration background, based on information from the SSS. At the school level, we controlled for the proportion of students with university educated parents and the proportion of students with a foreign background, based on administrative register information.

### Statistical method

The statistical method used was multilevel modelling. Two-level binary logistic regression models were estimated using the “meqrlogit” command in Stata, version 15 [29]. Results are presented as odds ratios (OR) with 95% Confidence Intervals (95% CI).

## Results

Descriptive statistics are presented in Table 1. In our sample, 29.8% of the students reported heavy episodic drinking and 12.3% reported problematic familial alcohol use. The sample consisted of about equal shares of boys and girls. Around two thirds lived with two parents in the same household and about a third did not. About two thirds had at least one parent with university education and 9% had lived in Sweden for less than ten years. Mean value, standard deviation, range of all school-level variables and possible scale range within brackets are presented at the bottom of Table 1.

**Table 1:** Descriptives

	n	%		
Student level				
Heavy episodic drinking	1405	29.8		
Problematic familial alcohol use				
No	4128	87.7		
Yes	581	12.3		
Gender				
Boy	2177	46.2		
Girl	2532	53.8		
Family structure				
Two-parent household	3025	64.2		
Other	1684	35.8		
Parental university education				
No or not known	1537	32.6		
At least one parent	3172	67.4		
Migration background				
≥ 10 years in Sweden	4285	91.0		
< 10 years in Sweden	424	9.0		

	Mean	SD	Min.	Max.
School level (scale points)				
Overall school ethos (0–28.9)	62.7	6.1	46.4	75.3
Sub-dimensions of ethos				
Staff stability (0–6.0)	8.8	1.3	4.7	11.7
Teacher morale (0–7.3)	15.8	1.7	12.0	19.3
Structure and order (0–9.0)	14.3	1.6	9.3	18.3
Student focus (0–5.1)	16.6	1.1	13.9	19.0
Academic atmosphere (0–4.8)	7.3	1.4	4.6	9.4
% students with at least one parent with university education (0–100)	52.0	25.1	7.0	86.3
% students with a foreign background (0–100)	40.9	21.4	6.0	95.7

n = 4709 students in 46 upper secondary schools

A series of two-level binary logistic regression analyses with heavy episodic drinking as the dependent variable are presented in Table 2. According to the empty model, 11.7% of the variation in heavy episodic drinking could be attributed to the school level. Model 1 includes student-level variables. Those who reported problematic familial alcohol use had a higher likelihood of reporting heavy episodic drinking compared with those who did not report problematic familial alcohol use (OR 1.36, 95% CI 1.12–1.65). Furthermore, girls were less likely than boys to have engaged in heavy episodic drinking (OR 0.77, 95% CI 0.67–0.88). Students not living with two parents in the same household had a greater likelihood of having reported heavy episodic drinking (OR 1.38, 95% CI 1.20–1.58), as were students who reported having at least one university-educated parent (OR 1.42, 95% CI 1.22–1.65). Students who had lived less than ten years in Sweden had a lower likelihood of reporting heavy episodic drinking (OR 0.31, 95% CI 0.22–0.44). Model 2 added overall school ethos, which showed a positive but not statistically significant association with heavy episodic drinking at the student level (OR 1.16, 95% CI 0.96–1.39). Next, the school proportion of students whose parent(s) had post-secondary education, and the school proportion of students with a foreign background were added (Model 3), but neither of these were statistically significant. Finally, in Model 4, we included the cross-level interaction between problematic familial alcohol use and overall school ethos, which was statistically significant (OR 0.79, 95% CI 0.65–0.97, p  =  0.022).

**Table 2:** Two-level binary logistic regression of heavy episodic drinking

	Empty model	Model 1		Model 2		Model 3		Model 4	
		OR	95% CI	OR	95% CI	OR	95% CI	OR	95% CI
Student level									
Problematic familial alcohol use									
No (ref.)		1.00	–	1.00	–	1.00	–	1.00	–
Yes		1.36**	1.12–1.65	1.36**	1.12–1.65	1.36**	1.12–1.65	1.38**	1.13–1.67
Gender									
Boy (ref.)		1.00	–	1.00	–	1.00	–	1.00	–
Girl		0.77***	0.67–0.88	0.77***	0.67–0.88	0.77***	0.67–0.89	0.77***	0.67–0.89
Family structure									
Two-parent household (ref.)		1.00	–	1.00	–	1.00	–	1.00	–
Other		1.38***	1.20–1.58	1.38***	1.20–1.59	1.38***	1.20–1.58	1.38***	1.20–1.59
Parental university education									
No or not known (ref.)		1.00	–	1.00	–	1.00	–	1.00	–
At least one parent		1.42***	1.22–1.65	1.41***	1.21–1.64	1.41***	1.21–1.65	1.41***	1.20–1.64
Migration background									
≥ 10 years in Sweden (ref.)		1.00	–	1.00	–	1.00	–	1.00	–
< 10 years in Sweden		0.31***	0.22–0.44	0.31***	0.22–0.44	0.31***	0.22–0.44	0.31***	0.22–0.44
School level									
Overall school ethos				1.16	0.96–1.39	1.15	0.94–1.40	1.19	0.97–1.45
% students with parents with post-secondary education						1.00	0.99–1.01	1.00	0.99–1.01
% students with a foreign background						1.00	0.99–1.01	1.00	0.99–1.01
Cross-level interaction									
Problematic familial alcohol use*								0.79*	0.65–0.97
Overall school ethos									
Intraclass Correlation (ICC) (%)	11.7***	9.5***		8.9***		8.8***		8.7***	

n  =  4709 students in 46 upper secondary schools

^***^p  <  0.001; **p  <  0.01; *p  <  0.05

To further assess the cross-level interactions between problematic familial alcohol use and different sub-dimensions of school ethos, we performed a set of analyses where we included one sub-dimension at a time as well as the cross-level interaction between the sub-dimension and problematic familial alcohol use. The estimates of the cross-level interactions from five separate two-level binary regression analyses are displayed in Table 3. The results show that for all sub-dimensions of school ethos except structure and order, there were statistically significant cross-level interactions with problematic familial alcohol use, demonstrating a weaker association with heavy episode drinking among students attending schools with higher levels of teacher-rated ethos.

**Table 3:** Cross-level interactions between problematic familial alcohol use and one sub-dimension of ethos at a time

Cross-level interactions	OR^a^	95% CI
Problematic familial alcohol use*	0.78*	0.65–0.95
Staff stability		
Problematic familial alcohol use*	0.79*	0.65–0.97
Teacher morale		
Problematic familial alcohol use*	0.95	0.77–1.18
Structure and order		
Problematic familial alcohol use*	0.80*	0.65–0.97
Student focus		
Problematic familial alcohol use*	0.79*	0.65–0.96
Academic atmosphere		

n  =  4709 students in 46 upper secondary schools

^a^Odds ratios from two-level binary logistic regression analyses of heavy episodic drinking. All models are adjusted for gender, family structure, parental university education, and migration background at the student level, and for the proportion of students with parents with post-secondary education and proportion of students with a foreign background at the school level

^*^p  <  0.05

## Discussion and conclusion

This study sheds light on the potential of the school to counteract problematic conditions in the family in relation to adolescent health risk behaviours. The findings corroborate previous research which has shown that parental drinking is linked with an increased likelihood of alcohol use in the offspring [9, 10, 20–22]. Furthermore, the study showed that teacher-rated school ethos had a moderating effect in that the association was weaker among students attending schools with higher teacher ratings of the ethos. This was true for overall school ethos and for four of five studied sub-dimensions of ethos: staff stability; teacher morale; student focus; and academic atmosphere. By suggesting that a strong school ethos has the potential to counteract poor conditions in the family in relation to student alcohol use, the result extends the findings of Olsson et al. [19]. The result is also in line with other available studies that have explored and found evidence of moderating effect of schools on youth problem behaviours [16, 30, 31]. However, it should be noted that the association between problematic familial alcohol use and heavy episodic drinking was not moderated by the sub-dimension structure and order for dealing with unwanted behaviour. Although this may seem somewhat counterintuitive, the result is in line with that of other studies [13, 23]. As suggested in these, articulated values for structure and order may not be effective means of reducing substance use in isolation from broader changes to the school environment. Hence, singled out from the other sub-dimensions of school ethos a school environment characterised by structure and order cannot be expected to compensate for risk laden parenting environments. Broader changes to the school ethos has to be put in place first.

Yet, taken together, the findings lend further support to the assumption that a favorable school environment may buffer against the potentially negative effects of problematic conditions in the family. Efforts placed at creating a school environment characterised by a strong school ethos in terms of staff stability, a strong teacher morale as well as a strong student and academic focus could thus be one way of avoiding problematic drinking patterns in the family to be transferred to adolescents. Making school leaders and policy makers aware of the potential of such broader interventions to prevent health risk behaviours, notably alcohol consumption among youth at risk, thus seems to be an important task. In addition, as suggested by Wiefferink et al. [32], looking beyond specific health behaviour directed policies, would also reduce the risk of teachers and school leaders becoming overloaded by extra-curricular activities and continuous innovations aimed at promoting student health and well-being.

## Limitations

The main limitation is the cross-sectional design of the study, which inhibits interpretations about causality with support in the data. Relatedly, we are not able to fully control for the selection of students into schools. There may be factors other than those accounted for in our study, that affect both students’ likelihood of attending schools with a certain level of ethos and their likelihood of engaging in heavy episodic drinking. There is also uncertainty regarding the validity of the measure of problematic familial alcohol use since information is obtained from students only, not parents or other family members. That data is based on self-reports may also, in particular in relation to questions about less socially acceptable behaviour, be associated with under- and over-reporting. However, the data has been sorted and questionnaires where for instance the reported amount of alcohol consumed was deemed as unreliable have been discarded [33]. Another limitation concerns the relatively low response rate among teachers (58%) and the scarce possibilities of checking any systematic bias among the non-responders. Finally, since the study was conducted in Stockholm municipality, the generalisability of the findings is limited.

## Data Availability

The data are not publicly available. The data from the Stockholm School Survey are available from Stockholm Municipality but restrictions apply to the availability of these data, which were used under license for the current study. Data from the Stockholm School Survey are however available from Stockholm Municipality upon reasonable request. Data from the Stockholm Teacher Survey can be applied for at the Department of Public Health Sciences, Stockholm University, Sweden.

## Abbreviations

95% CI: 95% confidence interval
ICC: Intraclass correlation
OR: Odds ratio
SSS: Stockholm School Survey
STS: Stockholm Teacher Survey
